# Novel method for retrieving Santorini duct stones from a patient with a rare chymotrypsin C variant combined with pancreas divisum

**DOI:** 10.1055/a-2447-2938

**Published:** 2024-11-13

**Authors:** Guangchao Li, Rui Ji, Fan Zhang, Ning Zhong, Yan-Qing Li, Peng Wang

**Affiliations:** 1 Department of Gastroenterology, Qilu Hospital of Shandong University, Jinan, China

An 18-year-old man with chronic pancreatitis presented with recurrent abdominal pain. He had undergone endoscopic retrograde pancreatography (ERP) 3 years earlier, which revealed incomplete pancreas divisum ( Fig. 1 ). Despite endoscopic sphincterotomy and stent placement, his abdominal pain persisted. Recent computed tomography showed acute peripancreatic effusion, calcification, and dilation of the Wirsung and Santorini ducts ( Fig. 2 ). Endoscopic ultrasound confirmed calcified stones impacted in the pancreatic duct ( Fig. 3 ). Subsequent ERP revealed the opaque stone impacted in the Santorini duct. Attempts were made to remove the stone via the major papilla, but the balloon failed to grab the stone ( Fig. 4 ). Intubation of the minor papilla was difficult. Pancreatoscopy was then used for further exploration.

**Fig. 1:**
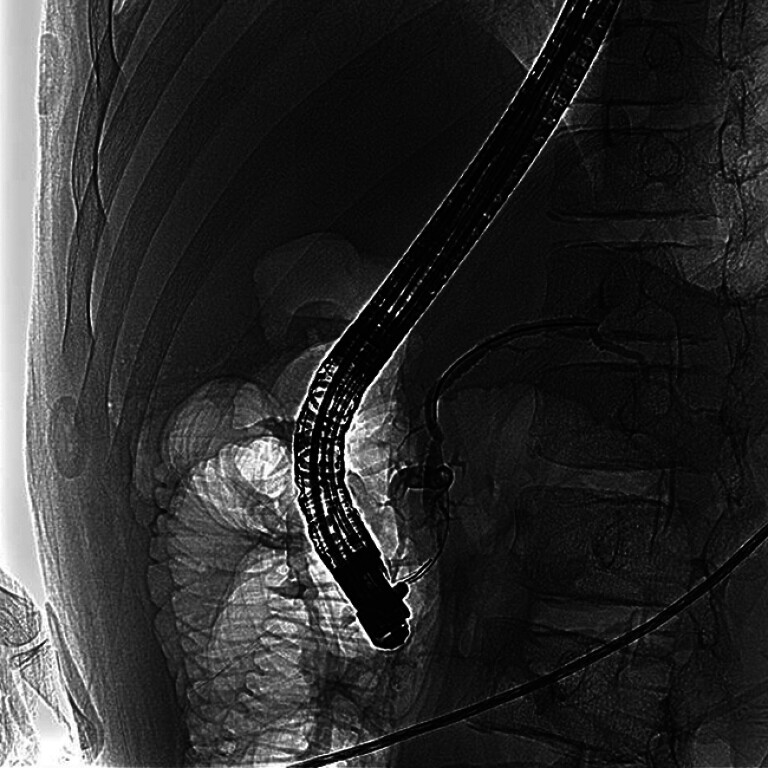
Previous endoscopic retrograde pancreatography revealed incomplete pancreas divisum.

**Fig. 2:**
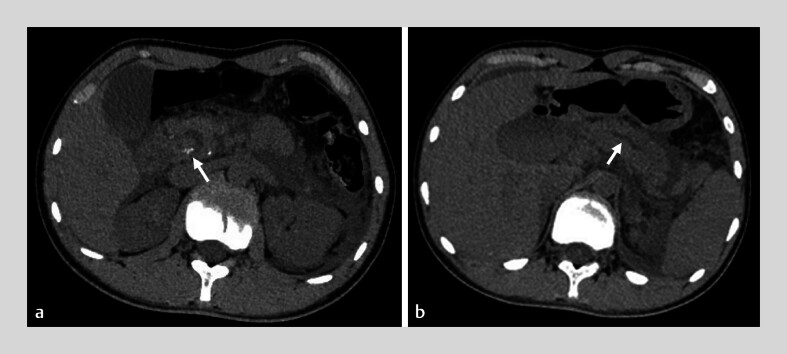
Computed tomography showed peripancreatic effusion, calcification, and dilation of the Wirsung and Santorini ducts (arrows).

**Fig. 3:**
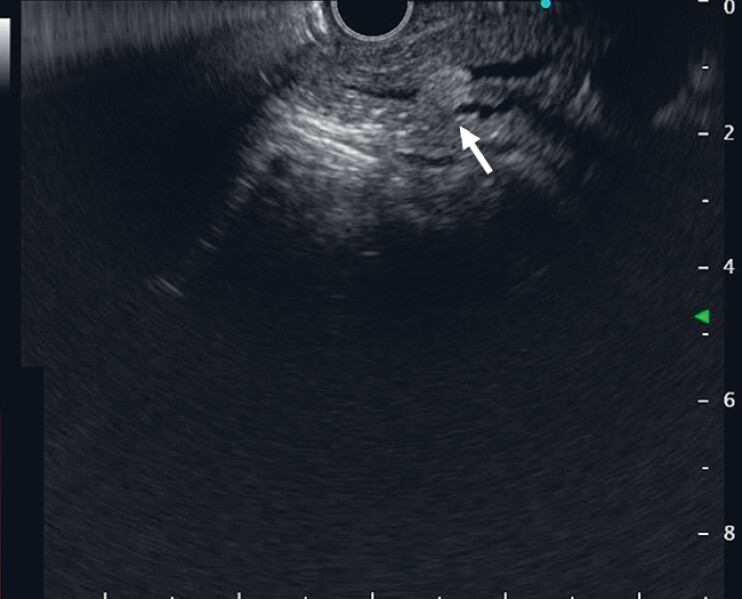
Endoscopic ultrasound showed the calcified stone (arrow) impacted in the pancreatic duct.

**Fig. 4:**
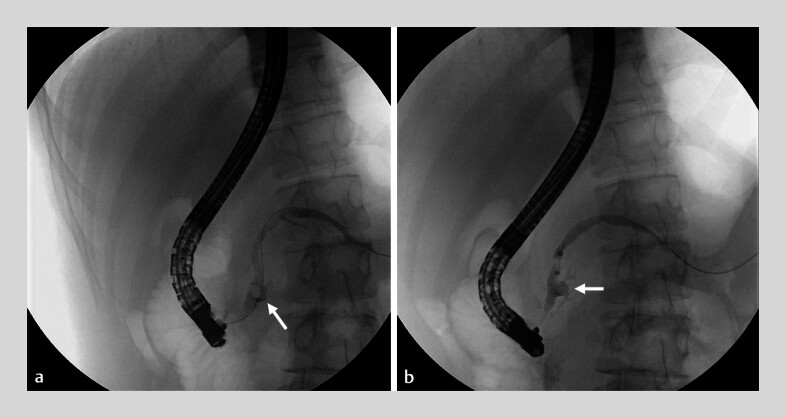
Endoscopic retrograde pancreatography revealed the opaque stone (arrows) impacted in the Santorini duct, but it could not be retrieved using a balloon.

**Fig. 5:**
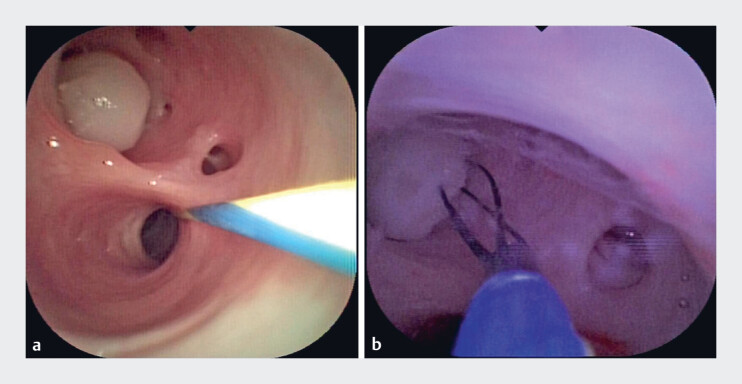
A four-wire retrieval basket captured and extracted the impacted stone under direct pancreatoscopic visualization.

A four-wire retrieval basket was inserted into the Santorini duct through pancreatoscopy and successfully captured and extracted the impacted stone under direct visualization ( Fig. 5 ). After the Santorini duct was cleared of stone fragments in a retrograde approach, intubation of the minor papilla was accomplished using the guidewire-assisted rendezvous technique and endoscopic minor papillotomy was performed.

The patient was discharged 5 days later without further discomfort ( Video 1 ). Genetic testing revealed a chymotrypsin C (CTRC) variant (719 G>A) from his mother, but no other family members had pancreatitis.

Pancreatoscopy and retrograde stone retrieval using a basket successfully removed stones from the Santorini duct in a patient with a rare chymotrypsin C variant combined with pancreas divisum.Video 1

Increasing evidence has revealed that pancreas divisum “acts as a partner of genetic mutations (mostly CFTR, SPINK1, and PRSS1)” or cofactor in causing pancreatitis 1 . The CTRC variant plays a significant role in calcific pancreatitis 2 . Remarkably, pancreas divisum combined with the CTRC variant is rare, making Santorini duct stones difficult to extract. Our case demonstrates that pancreatoscopy and retrograde retrieval of the stone using a basket under direct visualization is a novel and effective method for removing these stones when the minor papilla is difficult to intubate.

Endoscopy_UCTN_Code_CCL_1AZ_2AM

## Notes

Endoscopy E-Videos https://eref.thieme.de/e-videos
*E-Videos* is an open access online section of the journal *Endoscopy* , reporting on interesting cases and new techniques in gastroenterological endoscopy. All papers include a high-quality video and are published with a Creative Commons CC-BY license. Endoscopy E-Videos qualify for HINARI discounts and waivers and eligibility is automatically checked during the submission process. We grant 100% waivers to articles whose corresponding authors are based in Group A countries and 50% waivers to those who are based in Group B countries as classified by Research4Life (see: https://www.research4life.org/access/eligibility/ ). This section has its own submission website at https://mc.manuscriptcentral.com/e-videos .
